# The Effect of Complete Blood Count Timing on Lumbar Puncture Rates in Asymptomatic Infants Born to Mothers with Chorioamnionitis

**DOI:** 10.7759/cureus.3737

**Published:** 2018-12-16

**Authors:** Sadaf H Kazmi, Sean M Bailey, Pradeep V Mally, Sourabh Verma, William Borkowsky, Heather B Howell

**Affiliations:** 1 Pediatrics, New York University School of Medicine, New York, USA

**Keywords:** newborn, sepsis, complete blood count, white blood cell count, i/t ratio, lumbar puncture, chorioamnionitis, neonatal intensive care unit

## Abstract

Background

Maternal chorioamnionitis is a risk factor for sepsis but, often, these infants are asymptomatic at birth. Different markers for infections, such as the immature to total (I/T) white blood cell (WBC) ratio, are used to help determine which infants require lumbar punctures (LPs), in addition to blood cultures and antibiotics. The timing of when the complete blood count (CBC) is obtained may have some effect on the length of antibiotic treatment.

Aims

The purpose of this proof-of-concept study was to assess if obtaining a CBC at greater than four hours of life as compared to less than four hours of life has an impact on the incidence of LPs performed in asymptomatic, full-term infants undergoing evaluation for sepsis secondary to maternal chorioamnionitis.

Methods

We performed a retrospective study of full-term, asymptomatic infants admitted for sepsis evaluation secondary to maternal chorioamnionitis. Subjects were grouped based upon the timing of their initial CBC (early = < four hours of life or late = > four hours of life). The incidence of LPs, duration of antibiotic treatment, and length of hospitalization were compared between the groups.

Results

A total of 230 subjects were included in the study (early group = 124, late group = 106). Subjects in the late group underwent significantly fewer LPs than subjects in the early group, 5.7% vs. 22.6% (p<0.001). There was no difference in length of treatment or hospitalization.

Conclusions

Asymptomatic full-term infants undergoing evaluation for sepsis secondary to maternal chorioamnionitis are less likely to undergo an LP if their initial CBC is obtained at greater than four hours of life.

## Introduction

Neonatal sepsis is a major cause of morbidity and mortality. There is often an absence of clinical signs at the early stages of infection, or the presence of non-specific ones. Therefore, this frequently poses a clinical dilemma for physicians caring for these infants. Early onset sepsis (EOS) is defined by the Centers for Disease Control and Prevention (CDC) as blood and/or cerebrospinal spinal fluid (CSF) culture-proven infection occurring in the newborn at less than seven days of life [1]. The major risk factors for early-onset neonatal sepsis include preterm birth, prolonged rupture of membranes (PROM), maternal colonization with group B streptococcus, and maternal chorioamnionitis [2-3]. Maternal chorioamnionitis occurs in 1%-4% of all births in the US [4] and chorioamnionitis has been linked to 40% of cases of neonatal sepsis [5].

Asymptomatic infants are often treated empirically with antibiotics while undergoing an evaluation for sepsis based only on a diagnosis of chorioamnionitis in the infant’s mother. This is despite the fact that the incidence of EOS in this population remains low [6]. EOS is a rare disease, but with potentially devastating outcomes, if present [7]. A sepsis evaluation involves drawing blood cultures and starting infants on broad-spectrum antibiotics for a minimum of 48 hours. Currently, blood culture remains the gold standard test for sepsis, but it can be time-consuming, has limited sensitivity, and pathogens are only detected in approximately 25% of cases [8]. Therefore, the diagnosis of sepsis is often based on a clinical assessment in combination with laboratory markers, which have a limited diagnostic accuracy (low positive predictive value), such as white blood cell (WBC) indices, acute phase reactants, and heart rate variability [9-10].

A readily available screening marker for infection in the neonatal intensive care unit (NICU) is the complete blood count (CBC). The total WBC count has been found to have little value in diagnosis and to have a poor predictive accuracy [11-13]. Therefore, other subcomponents of the WBC are also analyzed, such as the immature to total (I/T) WBC ratio [14]. The timing of a CBC in relation to an infant’s hour of life after birth can influence test results. Recent studies have shown that waiting to obtain a CBC after four hours of life may be more accurate because mature and immature neutrophils require time for an inflammatory response [15-16]. In a study done by Newman et al. [17], the I/T ratio did provide some information when obtained in the first hour of life, but it was much more reliable in predicting infection when performed after four hours of life.

In newborn infants, the assessment of cerebrospinal fluid (CSF) for meningitis by lumbar puncture (LP) can also be part of a sepsis workup but has its associated risks [18-19]. Risks include possible infection, bleeding, and, rarely, damage to the spinal cord. In about 30% of patients, the LP is traumatic or inadequate, therefore, making the results difficult to analyze [20]. Wiswell et al. [21] determined that the incidence of bacterial meningitis is 0.25 per 1000 live births and occurs in about 30% of infants with culture-positive proven EOS [20]. In contrast, in infants that are being evaluated purely based on risk factors, rather than signs, the incidence of bacterial meningitis is extremely low [22-24]. Despite this, asymptomatic patients with elevated markers of infection may undergo a CSF assessment to rule out meningitis. The CSF results from these LPs can influence clinical decisions, including the length of antibiotic treatment, and, ultimately, the length of a patient’s hospital course.

At our institution, in 2012, there was a change in practice from obtaining a CBC immediately after birth to waiting until the infant was at least four hours old in asymptomatic infants admitted secondary to maternal chorioamnionitis. This practice change was based on data published showing that the value of a CBC in distinguishing infection dramatically increases after four hours of life [17]. The purpose of this proof-of-concept study was to assess if this practice change would result in a decrease in the number of LPs performed in this low-risk population. We hypothesized that obtaining a CBC at greater than four hours of life would lead to a reduction in the number of LPs. Our secondary outcome was to assess if a change in timing of the initial CBC would have any impact on the length of antibiotic treatment and the overall duration of hospital stay.

## Materials and methods

We conducted a retrospective cohort study of infants admitted to the NICU at New York University (NYU) Langone Health. The study was approved by the institutional review board. The eligible study population included any inborn infant greater than 37 weeks gestation who was admitted to the NICU for observation and evaluation for EOS secondary to maternal chorioamnionitis. Maternal chorioamnionitis was diagnosed by the obstetrician at the time of delivery, with a minimum requirement of maternal fever, and these mothers were treated with antibiotics.

Upon admission to the NICU, infants had a blood culture drawn and were started on ampicillin and gentamicin. CBCs were drawn either initially at birth with the blood culture or after four hours of life. Subjects underwent LPs if the I/T ratio was greater than 0.25 on the initial CBC. Repeat CBCs and C-reactive proteins (CRP) were drawn on some subjects to help determine the length of treatment, but not routinely in all subjects. Infants were excluded from the study if they showed any signs of sepsis during their hospitalization, including apnea, abnormal thermal regulation, respiratory distress, an abnormal neurologic exam, poor feeding, or abnormal blood glucose regulation.

Data including baseline infant and maternal demographics and prenatal and hospital course data were collected. Maternal prenatal data included various risk factors for neonatal infection, such as gestational age, PROM, maternal colonization with group B streptococcus, intrapartum antibiotics, and maternal chorioamnionitis. Infant hospital course data included a CBC with differential values, blood and CSF culture results, length of antibiotic treatment, the overall duration of hospital stay, and if they were readmitted after discharge.

The subjects were divided into two groups based upon the time that their initial CBC with differential was drawn. The early group consisted of infants whose initial CBC was obtained at less than four hours of life while the late group consisted of infants whose initial CBC was obtained at greater than four hours of life. A power calculation was performed, which determined that 220 subjects would be needed for this study. This was derived using a two-tailed method, an alpha error of 0.05, a power of 0.8, and a targeted 50% reduction in the incidence of LPs.

Statistical analyses were performed using SPSS 20.0 (IBM SPSS Statistics for Windows, Version 20.0. Armonk, NY, US: IBM Corp.) Data were expressed as means with standard deviations or medians with ranges. The two groups were compared using chi-square or student t-tests.

## Results

**Figure 1:**
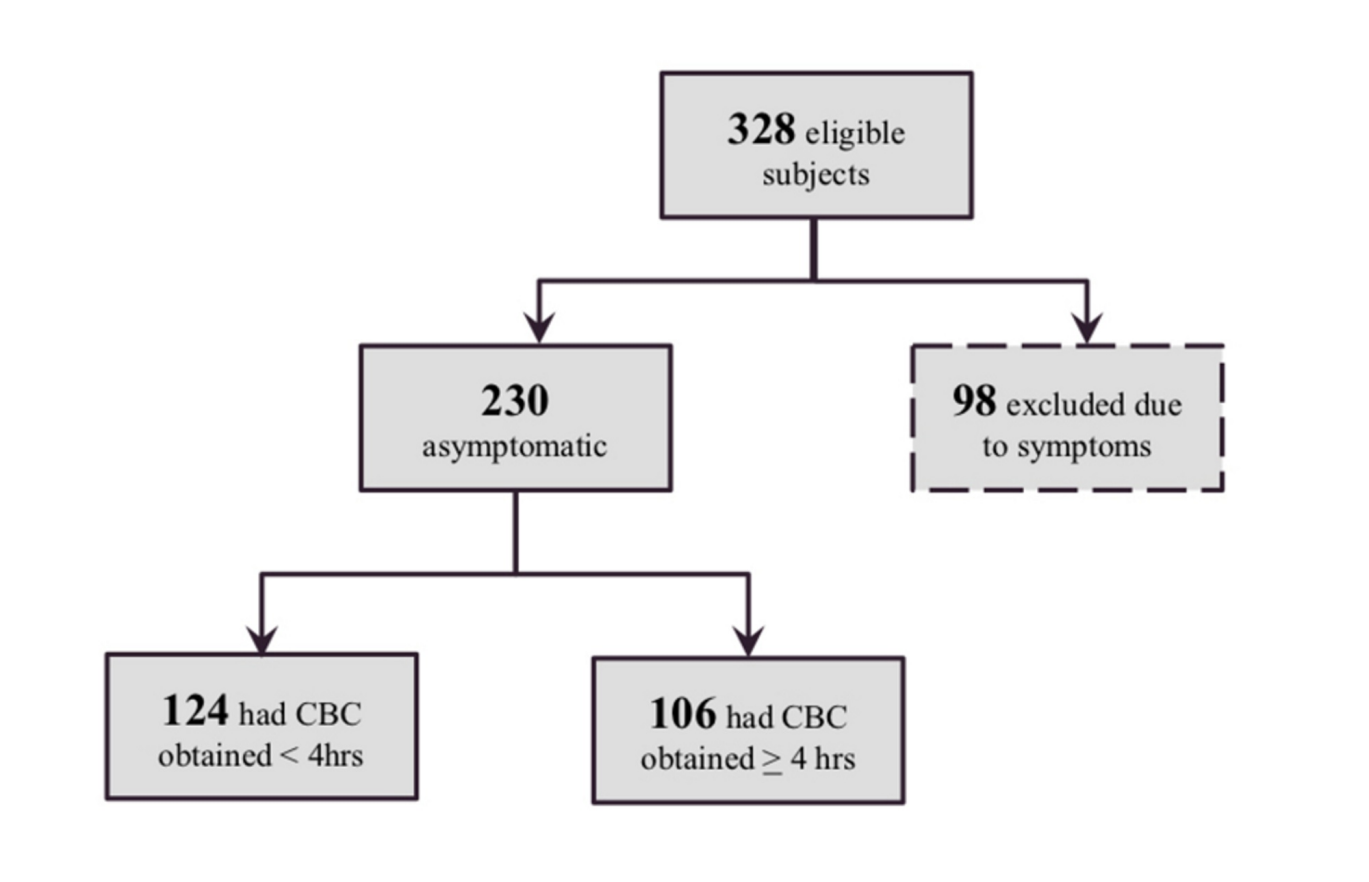
Study Population Attrition CBC = Complete Blood Count

There were 328 full-term infants admitted to the NICU at NYU Langone Health for sepsis assessment and evaluation secondary to maternal chorioamnionitis between January 2009 and October 2014. Ninety-eight subjects developed signs of possible sepsis during their hospitalization and were excluded. Of the 230 asymptomatic infants that made up our study population, 124 had their initial CBC with differential drawn at less than four hours of life and 106 had their initial CBC drawn at greater than four hours of life (Figure 1). On average, the CBC was obtained at 1 hour and 20 minutes in the early group and at 7 hours and 43 minutes in the late group.

Table 1 summarizes maternal and infant demographic data and select clinical characteristics. Maternal age was found to be older in the late group (p = 0.02) but, otherwise, there were no other significant differences between the two groups.

**Table 1:** Maternal and Infant Demographic Information and Clinical Characteristics SD = Standard Deviation; GBS = Group B Streptococcus

	Early Group (N=124)	Late Group (N=106)	p-value
Maternal Age (years), mean + SD	31.0 + 5.9	32.8 + 4.7	0.02
GBS positive, %	23.9%	16.0%	NS
Prolonged Rupture of Membranes, %	23.1%	25.4%	NS
Intra-partum Antibiotics, %	80.3%	83.9%	NS
Cesarian Section, %	35.0%	38.7%	NS
Male, %	53.2%	48.1%	NS
Birth Weight (grams), mean + SD	3365 + 482	3382 + 394	NS
Gestational Age (weeks), mean + SD	39.8 + 1.1	39.3 + 5.6	NS
APGAR 1 minute, median (25-75 percentile)	9 (8-9)	9 (8-9)	NS
APGAR 5 minute, median (25-75 percentile)	9 (9-9)	9 (9-9)	NS
Positive Pressure Ventilation, %	4.8%	4.7%	NS

The primary outcome compared the incidence of infants who underwent LP to assess CSF for infection (Table 2). Twenty-eight subjects (22.6%) in the early group underwent LP as compared with six subjects (5.7%) in the late group; this reduction was found to be statistically significant (p<0.001).

**Table 2:** Comparison of Mean I/T Ratios and Percent of Lumbar Punctures I/T = Immature to Total; SD = Standard Deviation; LP = Lumbar Puncture

	Early Group (N=124)	Late Group (N=106)	p-value
I/T Ratio, mean + SD	0.25 + 0.18	0.15 + 0.14	<0.001
LPs Performed, n (%)	28 (22.6)	6 (5.7)	<0.001

The mean I/T ratio for each group was calculated and was found to be significantly lower (p<0.001) in the late group (Table 2). The mean I/T ratio (0.47 +0.18) in the 33 subjects who underwent LP was found to be significantly higher than the mean I/T ratio (0.16 +0.12) of the 197 subjects who did not undergo LP (p<0.001). A further assessment was done to compare the I/T ratios in the subjects who underwent LP in both groups. We found that those subjects who underwent LP, the I/T ratio for the subjects in the early group (0.48 +0.18) was not significantly different than the I/T ratio of those subjects in the late group (0.43+0.17).

**Figure 2:**
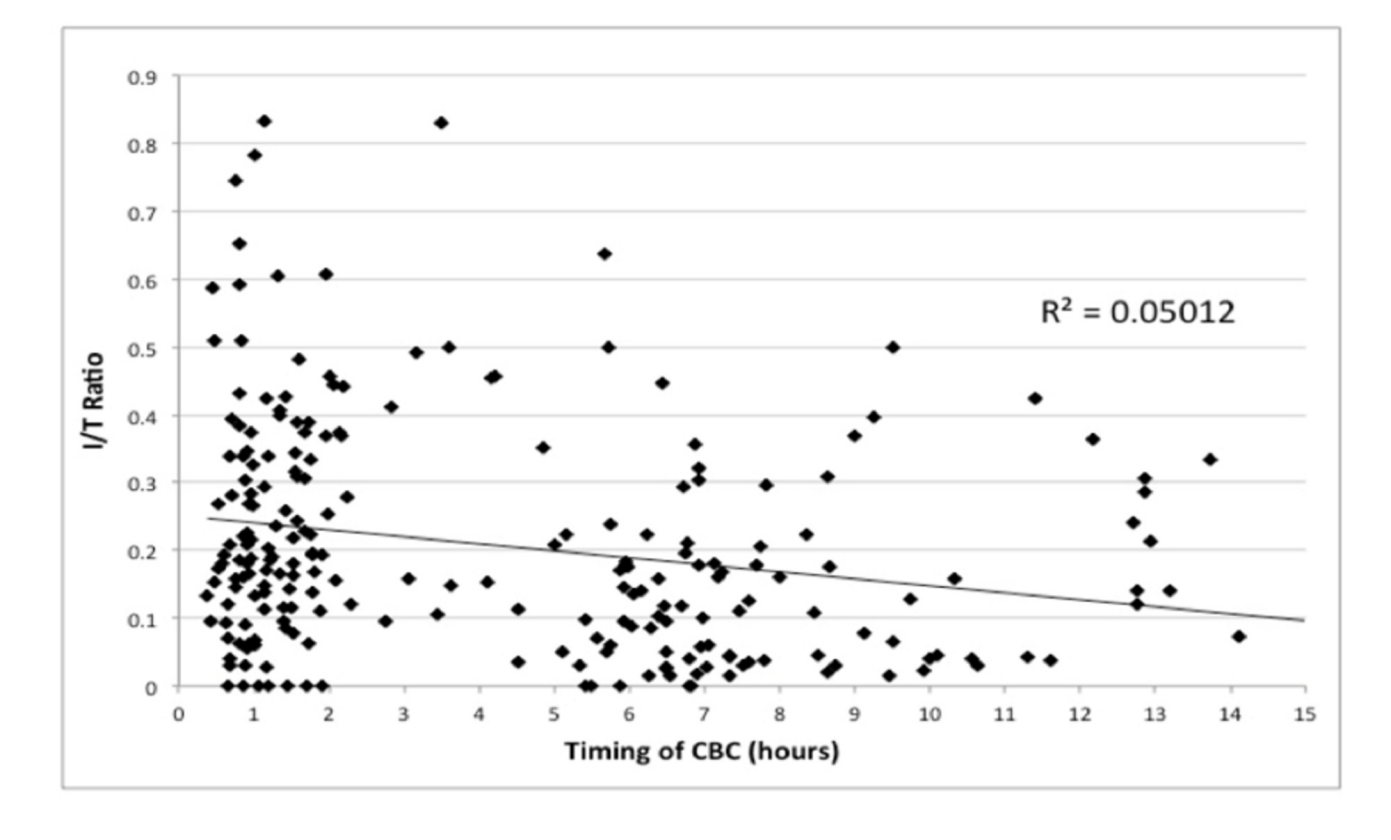
I/T Ratio vs. Timing of CBC, p< 0.001 I/T = Immature to Total; CBC = Complete Blood Count

We performed a correlation analysis examining the I/T ratio against the time at which the CBC was drawn (Figure 2). A downward trend in the I/T ratio over time was noted, with an R^2^ value of 0.05 and a significant p-value of 0.001.

We assessed our secondary outcomes of duration of antibiotic treatment and length of hospitalization. As shown in Table 3, there was no statistical difference found between the early group and the late group in either of these measures. There were no positive blood or CSF cultures from any of the subjects in this study.

**Table 3:** Comparison of Mean Duration of Antibiotics Treatment and Duration of Hospitalization SD = Standard Deviation

	Early Group (N=124)	Late Group (N=106)	p-value
Duration of Antibiotics (days), mean + SD	2.7 + 2.5	2.5 + 1.4	NS
Duration of Hospitalization (days), mean + SD	2.9 + 2.4	2.6 + 1.5	NS

Lastly, we looked at the rate of readmission back to NYU Langone Health for all subjects in the study one-month post-discharge. One subject from the late group was readmitted within one week of discharge for a fever at home but was afebrile upon arrival to the emergency room (ER) and all blood, urine, and CSF cultures were negative during the readmission.

## Discussion

EOS in neonates can be life-threatening if not properly recognized and treated. The implementation of intrapartum antibiotic prophylaxis for the prevention of group B streptococcal disease has reduced the overall incidence, which is estimated in the United States to be 0.77 to 1 per 1,000 live births [25]. Despite this reduction, early onset neonatal sepsis remains a serious problem. The American Academy of Pediatrics’ recommendations utilize a perinatal risk factor-based evaluation for newborns and empiric treatment in asymptomatic infants [26]. This practice results in empiric antibiotic therapy in many uninfected infants. In this single-center proof-of-concept study, we demonstrated that obtaining a CBC at greater than four hours of life is associated with a reduction in the number of LPs that asymptomatic infants undergo during a rule out of sepsis when a mother is diagnosed with maternal chorioamnionitis. There was no difference seen in the length of treatment or hospitalization.

Maternal chorioamnionitis remains an important perinatal risk factor for EOS. Clinical maternal chorioamnionitis is defined as maternal fever (>100.4 degrees Fahrenheit) with at least two of the following: maternal tachycardia, fetal tachycardia, foul-smelling discharge, uterine tenderness, or maternal leukocytosis [4,27]. Infants born to mothers diagnosed with chorioamnionitis are often admitted to the NICU for evaluation, observation, and empiric antibiotic therapy to prevent EOS. Since symptoms of sepsis in the newborn can be absent at the early stages, or non-specific, these patients pose a challenge to pediatricians and neonatologists. The I/T ratio has been found to have the best sensitivity of all the neutrophil indices, including absolute neutrophil count and absolute band count. A single determination has a poor predictive accuracy but a very high negative predictive accuracy [28]. The I/T ratio is less than 0.22 in 96% of healthy infants [28-29]. Previous studies have shown that the value of a CBC with differential to distinguish the presence of infection dramatically increases after four hours of life [15-17].

Due to a practice change in our NICU, we were able to compare asymptomatic, full-term infants admitted to the NICU secondary to maternal chorioamnionitis who had their initial CBC obtained at less than four hours of life to infants who had their initial CBC obtained at greater than four hours of life. The two study groups, when compared, were found to be similar except for maternal age, which we do not think is clinically relevant in this study. Our results showed a significant reduction in the incidence of infants who underwent LP to assess the CSF if the initial CBC was drawn at greater than four hours of life. There was a significant difference in the mean I/T ratios between the two groups, suggesting that, in this study, clinicians relied on this ratio to guide their decision regarding whether to pursue a CSF evaluation for meningitis. An I/T ratio of greater than 0.25 prompted a CSF evaluation in our subjects. We also found that the I/T ratio amongst the infants who underwent LPs to be comparable in the two groups. This supports the notion that clinicians are more likely to evaluate for meningitis in infants with an elevated I/T ratio, independent of the timing of the initial CBC. Therefore, the significant decrease in the incidence of LPs we found between the two groups likely reflects the decreased I/T ratio seen in the late group.

When the I/T ratio was trended over time, there appeared to be an initial spike immediately after birth, which then decreased over time. This correlation was found to be statistically significant, but there was still a high level of variability seen amongst the data points. The trend line at four hours of life was noted to cross at an I/T ratio around 0.25, strengthening the use of this value as a cut-off to perform a CSF evaluation in our study.

There was no difference between the two groups in terms of the length of antibiotic treatment or duration of hospitalization. This finding may reflect the influence of cultures, as there were no positive blood or CSF cultures in either group. Clinicians may utilize the CBC with differential as an additional source of information, but in asymptomatic infants, the culture results have a stronger influence on determining the length of antibiotic treatment and, ultimately, the length of stay.

The first limitation of our study was that we did not account for other laboratory markers that may be utilized in the evaluation for sepsis. Inflammatory markers, such as a CRP, erythrocyte sedimentation rate, or procalcitonin, are variably used to aid in clinical decision-making regarding sepsis. We did not evaluate the use or influence of these markers in our study population because this is not standard practice at our institution. We also did not account for subsequent CBC values on individual subjects and how a trend over time may also affect treatment decisions. Another limitation of this study was the limited one-month post-discharge follow-up data for the population. All infants remained without signs of infection during their hospitalization and had negative culture results. Only one subject was readmitted to NYU Langone Health, and all cultures remained negative during the admission. Due to the retrospective nature of the study, we do not have information on whether any of our subjects were readmitted or visited an emergency room at a different hospital. Lastly, we did not collect data about hyperbilirubinemia and the need for phototherapy, which may have affected the length of hospitalization.

## Conclusions

To conclude, when comparing two groups of asymptomatic, full-term newborns undergoing evaluation for sepsis secondary to maternal chorioamnionitis, we found that obtaining an initial CBC at greater than four hours of life is associated with a significant decrease in the mean I/T ratio and a significant decrease in the rate of LPs performed, and this appears to be a safe practice. There was no difference between the two groups in the length of antibiotic treatment or duration of hospitalization. Future studies are needed to establish a more comprehensive assessment of these newborns with the goal of identifying those truly at risk for early EOS and avoiding the overuse of antibiotic therapy and LPs in the uninfected.
